# Racial and Ethnic Disparities in Fetal Deaths — United States, 2015–2017

**DOI:** 10.15585/mmwr.mm6937a1

**Published:** 2020-09-18

**Authors:** Shannon M. Pruitt, Donna L. Hoyert, Kayla N. Anderson, Joyce Martin, Lisa Waddell, Charles Duke, Margaret A. Honein, Jennita Reefhuis

**Affiliations:** ^1^National Center on Birth Defects and Developmental Disabilities, CDC; ^2^Oak Ridge Institute for Science and Education, Oak Ridge, Tennessee; ^3^National Center for Health Statistics, CDC; ^4^March of Dimes, White Plains, NY.

The spontaneous death or loss of a fetus during pregnancy is termed a fetal death. In the United States, national data on fetal deaths are available for losses at ≥20 weeks’ gestation.* Deaths occurring during this period of pregnancy are commonly known as stillbirths. In 2017, approximately 23,000 fetal deaths were reported in the United States (*1*). Racial/ethnic disparities exist in the fetal mortality rate; however, much of the known disparity in fetal deaths is unexplained (*2*). CDC analyzed 2015–2017 U.S. fetal death report data and found that non-Hispanic Black (Black) women had more than twice the fetal mortality rate compared with non-Hispanic White (White) women and Hispanic women. Fetal mortality rates also varied by maternal state of residence. Cause of death analyses were conducted for jurisdictions where >50% of reports had a cause of death specified. Still, even in these jurisdictions, approximately 31% of fetal deaths had no cause of death reported on a fetal death report. There were differences by race and Hispanic origin in causes of death, with Black women having three times the rate of fetal deaths because of maternal complications compared with White women. The disparities suggest opportunities for prevention to reduce the U.S. fetal mortality rate. Improved documentation of cause of death on fetal death reports might help identify preventable causes and guide prevention efforts.

CDC used the 2015–2017 fetal death data files and birth certificates available from the National Vital Statistics System. Records were restricted to exclude fetal deaths occurring to non-U.S. residents and those of <20 weeks’ gestation as determined by the obstetric estimate of gestational age at delivery (*3*). Data from all 50 states and the District of Columbia were used to calculate fetal mortality rates. Cause of death was examined in jurisdictions that used the 2003 revision of the standard fetal death report^†^ and where >50% of reports had a specified cause of death.

Fetal mortality rates are expressed as the number of fetal deaths per 1,000 live births plus fetal deaths. Rates were calculated nationally and by mothers’ state of residence, race and Hispanic origin, age, and multiple-gestation pregnancy. Causes of death were reported on the fetal death report according to codes from the *International Classification of Diseases, Tenth Revision *(ICD-10). Codes for cause of death were categorized into 45 ranked causes of death, from which the selected causes were drawn (*4*). The five most common cause of death categories for the reporting jurisdictions^§^ were examined by maternal race and Hispanic origin. Using a Poisson model, 95% confidence intervals (CIs) around the fetal mortality rate and crude rate ratios (RRs) were calculated. Data analysis was completed using SAS software (version 9.4; SAS Institute).

Overall, during 2015–2017, the U.S. fetal mortality rate was 6.0 per 1,000 live births and fetal deaths (Figure 1). Among Black women, the fetal mortality rate (11.2; 95% CI = 11.1–11.4) was more than twice that among White women (5.0; 95% CI = 5.0–5.1) and Hispanic women (5.1; 95% CI = 5.0–5.2). The fetal mortality rate among mothers aged <20 years (7.4) was 30% higher than that among mothers aged 20–39 years (5.7; RR = 1.3; 95% CI = 1.2–1.3), and the rate among mothers aged >40 years (10.0) was also significantly higher than that among mothers aged 20–39 years (RR = 1.8; 95% CI = 1.7–1.8). Fetal mortality among women who had multiple-gestation pregnancies (13.7) was more than twice that of mothers carrying singletons (5.7; RR = 2.4; 95% CI = 2.4–2.5).

**FIGURE 1:**
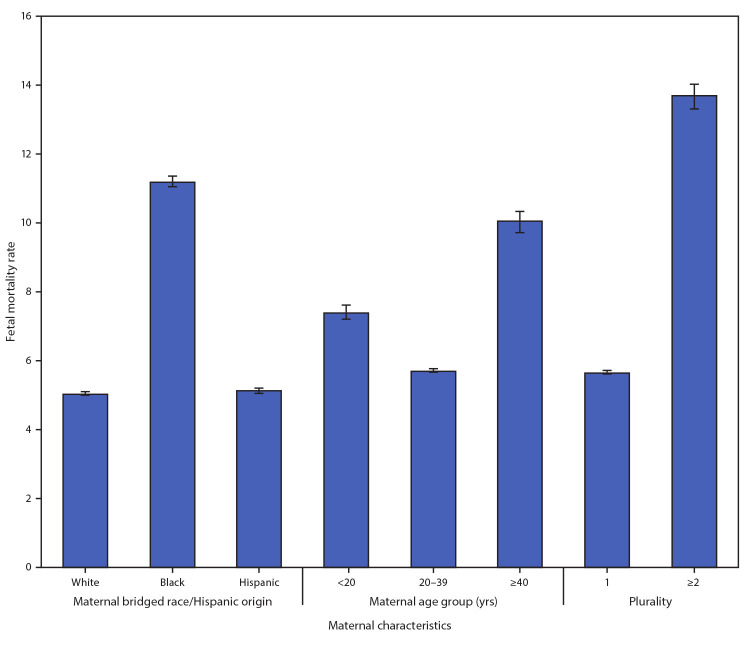
Fetal mortality rates,* by selected maternal characteristics^†^ — United States,^§^ 2015–2017 * Fetal deaths per 1,000 births plus fetal deaths. ^†^ Black women and White women were non-Hispanic; Hispanic women could be of any race. ^§^ Maternal bridged race/Hispanic origin excludes Rhode Island in 2015 because the state was unable to provide data on maternal race and Hispanic origin on the fetal death report.

The fetal mortality rate varied by U.S. state. Overall, rates were higher in the southern United States (Figure 2); Alabama reported the highest state-level fetal mortality rate among White women (6.9; 95% CI = 6.4–7.4) and Hispanic women (7.0; 95% CI = 5.8–8.6). Fetal mortality rates among Black women exceeded 16 per 1,000 in New Jersey (17.3; 95% CI = 16.1–18.7), West Virginia (16.8; 95% CI = 11.8–23.8), and Mississippi (16.3; 95% CI = 15.2–17.5).

**FIGURE 2:**
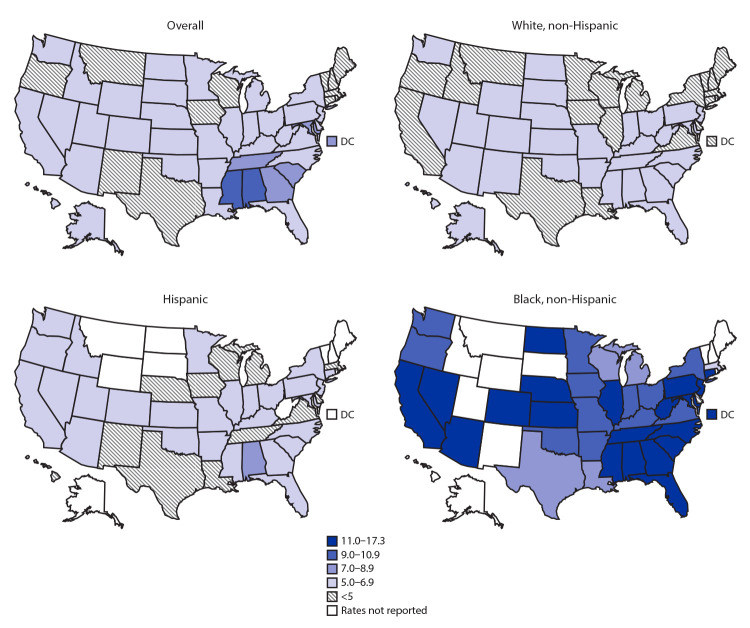
Fetal mortality rates,*^,^^†^ by state — United States, 2015–2017 **Abbreviation:** DC = District of Columbia. * Fetal deaths per 1,000 live births plus fetal deaths. † Rates for states that reported fewer than 20 fetal deaths are not presented. The rate for Rhode Island is not presented because the state was unable to provide data on maternal race and Hispanic origin on the fetal death report in 2015.

Overall, 31% of fetal death reports had an unspecified cause of death. This was similar among Black, White, and Hispanic mothers. In the selected reporting jurisdictions, the five most common cause of fetal death categories were 1) complications of placenta, cord, and membrane; 2) maternal complications of pregnancy; 3) congenital malformations, deformations, and chromosomal abnormalities; 4) maternal conditions that might be unrelated to present pregnancy; and 5) syndrome of infant of diabetic mother and neonatal diabetes mellitus (Figure 3). The cause of death varied by maternal race and Hispanic origin. Among Black mothers, the rate of having a fetal death attributable to maternal conditions that might be unrelated to the present pregnancy was substantially higher than the rate among White mothers (1.4 versus 0.4; RR=3.4; 95% CI = 3.2–3.6), as was the rate of a fetal death attributable to maternal complications of pregnancy (1.8 versus 0.6; RR=3.1; 95% CI = 2.9–3.2). Compared with White mothers, Black mothers had elevated rates of fetal death attributable to syndrome of infant of a diabetic mother and neonatal diabetes mellitus (0.3 versus 0.1; RR = 2.8; 95% CI = 2.4–3.2); fetal death of unspecified cause (3.3 versus 1.6; RR = 2.0; 95% CI = 1.9–2.1); and fetus affected by complications of placenta, cord, and membranes (2.7 versus 1.4; RR = 2.0; 95% CI = 1.9–2.0). Compared with White mothers, Hispanic mothers had increased rates of fetal death attributable to maternal complications of pregnancy (0.8 versus 0.6; RR = 1.3; 95% CI 1.2–1.4) and syndrome of infant of a diabetic mother and neonatal diabetes mellitus (0.2 versus 0.1; RR = 2.1; 95% CI 1.8–2.4). No significant racial/ethnic differences in fetal mortality attributable to congenital malformations were identified.

**FIGURE 3:**
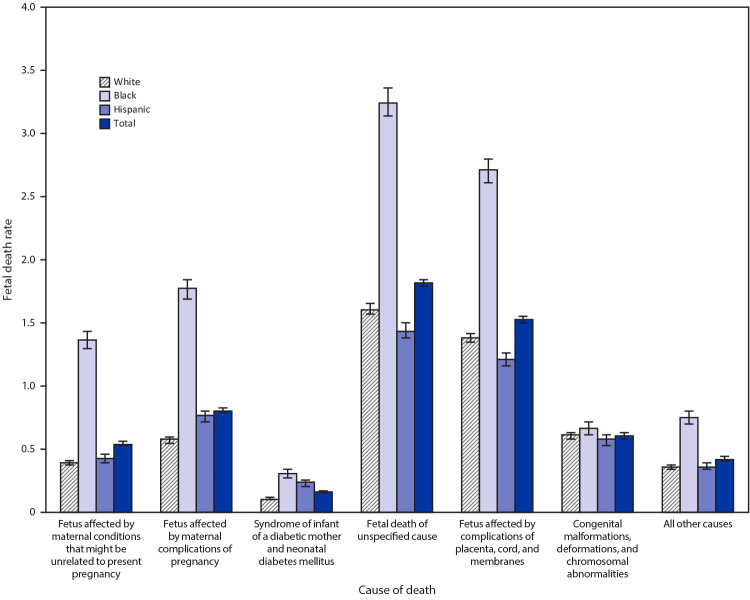
Fetal mortality rates,* by cause of death categories and maternal race/ethnicity^†^ among states where >50% of fetal deaths had a documented cause^§^,^¶^ — United States,** 2015–2017 * Deaths per 1,000 live births plus fetal deaths. ^†^ White women and Black women were non-Hispanic; Hispanic women could be of any race. ^§^ 2015: 39 states and the District of Columbia. Excludes California, Connecticut, Georgia, Mississippi, New Jersey, New York, North Carolina, North Dakota, Tennessee, West Virginia, and Wisconsin. 2016: 38 states and the District of Columbia. Excludes California, Connecticut, Georgia, Hawaii, Mississippi, New Jersey, New York, North Dakota, Tennessee, Vermont, West Virginia, and Wisconsin. 2017: 38 states and the District of Columbia. Excludes California, Connecticut, Georgia, Michigan, Mississippi, New York, North Dakota, Rhode Island, Tennessee, Vermont, Virginia, and Wisconsin. ^¶^ Thirty-one percent of records are assigned to an unspecified cause of death. If reporting or diagnostic improvements resulted in more specified causes of death, fetal mortality rates for the cause of death categories could change markedly. These potential changes may differ by race/Hispanic origin. ** Excludes Rhode Island in 2015 because the state was unable to provide data on maternal race and Hispanic origin on the fetal death report.

## Discussion

Fetal deaths in the United States are disproportionately higher among Black women than among White women; this racial disparity has been well-documented (*2*) and persistent (*5*). Other factors that increase the risk for fetal death include maternal age <20 or >40 years, and multiple-gestation pregnancy (*2*). This report also indicates variation in the fetal mortality rate among states; however, Black women experience increased fetal death rates nationwide. Although the reporting area differs, the most common causes of fetal death were similar to those reported previously (*6*). Findings from this report indicate that fetal mortality rates for all selected cause of death categories were higher among Black women than among White women, with the exception of congenital malformations, the rate of which was similar among all racial/ethnic groups examined. Rates of fetal mortality attributed to maternal complications of pregnancy and syndrome of infant of diabetic mother and neonatal diabetes mellitus were also increased among Hispanic women compared with those among White women.

The underlying reasons for the observed racial/ethnic disparities in fetal deaths are not fully understood. Some factors that might contribute to these disparities include differences in maternal preconception health, socioeconomic status, access to quality health care, stress, and racism, including institutional bias (*5*). There are opportunities for prevention of fetal deaths (*7*). Improvements in preconception health and prenatal care for Black women has the potential to reduce the disparity in fetal mortality rates (*5*,*8*); however, the lack of complete information on causes of fetal death has made it difficult to design and implement prevention strategies (*9*).

This findings in this report are subject to at least two limitations. First, because cause of fetal death is not available for states that do not use the 2003 revision of the fetal death report, and because jurisdictions where <50% of reports specified a cause of death were not included, presenting cause of death data nationwide was not possible. Therefore, this report is not nationally representative. Second, even in jurisdictions where >50% of reports specified a cause of death, nearly one third of records still lacked an informative cause. An improvement in reporting or diagnosis that resulted in fewer reports with unspecified causes would likely change the rate for other cause of death categories.

The U.S. fetal mortality rate has been relatively stable since 2006 (*10*), but racial/ethnic disparities persist and are demonstrated in four of the five most common cause of fetal death categories. Racial/ethnic disparities in causes of death could inform opportunities to reduce the U.S. fetal mortality rate. Results from this analysis suggest that reporting of causes of fetal deaths on fetal death reports could be improved. Given the racial/ethnic disparities in prevalence of fetal death and the incompleteness of many fetal death reports, opportunities for further research into preventable causes of fetal death are still to be determined.

**Summary**

**What is already known about this topic?**

Approximately 23,000 fetal deaths occurred in the United States in 2017. Data from the National Vital Statistics System show racial/ethnic disparities in fetal mortality.

**What is added by this report?**

During 2015–2017, the fetal mortality rate among non-Hispanic Black women was more than twice that among non-Hispanic White women and Hispanic women. Fetal mortality rates varied by state and cause of death category. The rate of fetal death attributable to maternal complications among non-Hispanic Black women was three times that among White women.

**What are the implications for public health practice?**

Racial/ethnic disparities in prevalence of fetal death suggest opportunities to reduce the U.S. fetal mortality rate. Improved documentation of causes of fetal death might help guide prevention efforts.

## Footnotes

* https://www.cdc.gov/nchs/data_access/vitalstatsonline.htm.

† https://www.cdc.gov/nchs/data/dvs/fetal_death_edit_specifications.pdf.
